# Insights into a novel nuclear function for Fascin in the regulation of the amino-acid transporter SLC3A2

**DOI:** 10.1038/srep36699

**Published:** 2016-11-07

**Authors:** Amine Saad, Krikor Bijian, Dinghong Qiu, Sabrina Daniela da Silva, Maud Marques, Chia-Hao Chang, Hassan Nassour, Dindial Ramotar, Sambasivarao Damaraju, John Mackey, Tarek Bismar, Michael Witcher, Moulay A. Alaoui-Jamali

**Affiliations:** 1 Departments of Medicine, Oncology, Pharmacology and Therapeutics , Segal Cancer Centre and Lady Davis Institute for Medical Research, Sir Mortimer B. Davis-Jewish General Hospital, 3755 Cote-Ste Catherine, Montreal, Quebec, H3T 1E2, Canada; 2 Otolaryngology-Head and Neck Surgery, Faculty of Medicine, McGill University, 3655 Promenade Sir William Osler, Montreal, Quebec, H3G 1Y6, Canada; 3 Maisonneuve-Rosemont Hospital, Research Center, University of Montreal, Immunology and Oncology, Montreal, Quebec, H1T 2M4, Canada; 4 Departments of Laboratory Medicine and Pathology, University of Alberta, Cross Cancer Institute, 11560 University Avenue, Edmonton, Alberta, T6G 1Z2, Canada; 5 Department of Oncology, University of Alberta, Cross Cancer Institute, 11560 University Avenue, Edmonton, Alberta, T6G 1Z2, Canada; 6 Departments of Pathology & Laboratory Medicine and Oncology, Southern Alberta Cancer Institute, University of Calgary, Alberta, Canada

## Abstract

Fascin 1 (FSCN1) is a cytoskeleton-associated protein recognized to function primarily in the regulation of cytoskeleton structure and formation of plasma membrane protrusions. Here we report a novel nuclear function for Fascin 1. Biochemical studies and genome wide localization using ChIP-seq identified phosphorylated Fascin 1 (pFascin) in complexes associated with transcription and that it co-localizes with histone H3 Lys4 trimethylation (H3K4me3) on chromatin. Gene expression profiling identified genes affected by Fascin 1 including SLC3A2, a gene encoding for a plasma membrane transporter that regulates intracellular amino acid levels. RbBP5, a subunit of the H3K4 histone methyltransferase (HMT) complex was found to interact with Fascin 1 supporting its role in H3K4me3 establishment at target genes. Moreover, we show that changes to SLC3A2 levels affect amino acid-mediated mTORC1 activation. These results reveal that Fascin 1 has a yet undiscovered nuclear function as an epigenetic modulator of genes essential for amino acid metabolism.

The integrity of the cell cytoskeleton is essential for multiple physiological functions such as the maintenance of tissue homeostasis, including cell shape and polarity, cell contractility, division, and locomotion, as well as intracellular trafficking of signaling molecules. The regulation of the cell cytoskeleton compartment is mediated by a collective set of actin binding and cross-linking proteins, which mediate branching of actin filaments into tightly organized networks where actin filaments become connected by entanglements, cross-linking, bundling, and via binding to motor proteins^1^. A major class of actin bundling proteins is represented by the Fascin family, which comprises Fascin-1, -2, and -3. Fascin 1 (henceforth mentioned as Fascin) is the most ubiquitous compared to Fascin 2 and Fascin 3, which are restricted to retina photoreceptor cells and the testis, respectively. Fascin protein encompasses an actin-binding domain (ABD) at the N-terminus^2^, a second ABD predicted at the C-term portion and a well-characterized phosphorylation site at the amino-terminal actin binding site (S39)^2^,^3^. Phosphorylation at this site, mediated by kinases such as PKCα, inhibits Fascin’s ability to bind actin and to form bundles required for the assembly of protrusions in migratory cells^3^.

Fascin is primarily expressed within the nervous system and in mature dendritic cells and is mainly absent from normally differentiated epithelium. In pathological conditions such as cancer, Fascin is upregulated in a variety of cancers, including ovarian, oesophageal, colorectal and breast carcinomas^4^,^5^,^6^,^7^, and in many instances an association between its overexpression and poor patient prognosis has been observed^8^,^9^. In this context, migrastatin, a molecule reported earlier to interfere with cancer metastasis^10^, was found to target Fascin in part via inhibition of its ability to bind actin^11^. Fascin has also been shown to mediate dendritic cell resistance to listeria infection^12^ and to be associated with other cancer-associated mechanisms, including tumor self-seeding concept^13^. How this actin bundling protein can impact such disparate cellular processes that have not been elucidated at the molecular level. A more recent paper by Groen *et al*., suggests the concept of a non-actin function of Fascin in Drosophila possibly in the maintenance of nucleolar architecture^14^. However, the magnitude of nuclear functions associated with nuclear Fascin remains largely unknown.

In this study, we identified a novel nuclear function for Fascin in transcriptional regulation. We used alternative methodologies to establish the nuclear localization of Fascin 1, enriched for its phosphoryalted form. Computational and mutational analysis identified a region surrounding the serine 39 (S39) phospho-residue of Fascin as responsible for its nuclear translocation. Co-immunoprecipitation and size exclusion chromatography revealed that nuclear phosphorylated Fascin associates with the RNA polymerase II (referred to as Pol2) complex. Gene expression microarray studies allowed us to identify target genes under direct Fascin regulation. ChIP-seq and ChIP experiments showed that pFascin colocalized with H3K4me3 accumulation across the genome and facilitates the deposition of this histone modification at the promoter of the target genes. Finally, we show that pFascin interacts specifically with the H3K4 methyltransferase core subunit RbBP5. Moreover, we report that the changes mediated by Fascin at the SLC3A2 locus potentiate mTORC1 activation in response to amino acids. To our knowledge, this is the first report of Fascin involvement in histone methylation and transcriptional regulation. These findings open novel avenues to explore the molecular function of Fascin in physiological and diseased conditions.

## Results

### Fascin is a nuclear protein

**Figure 1:**
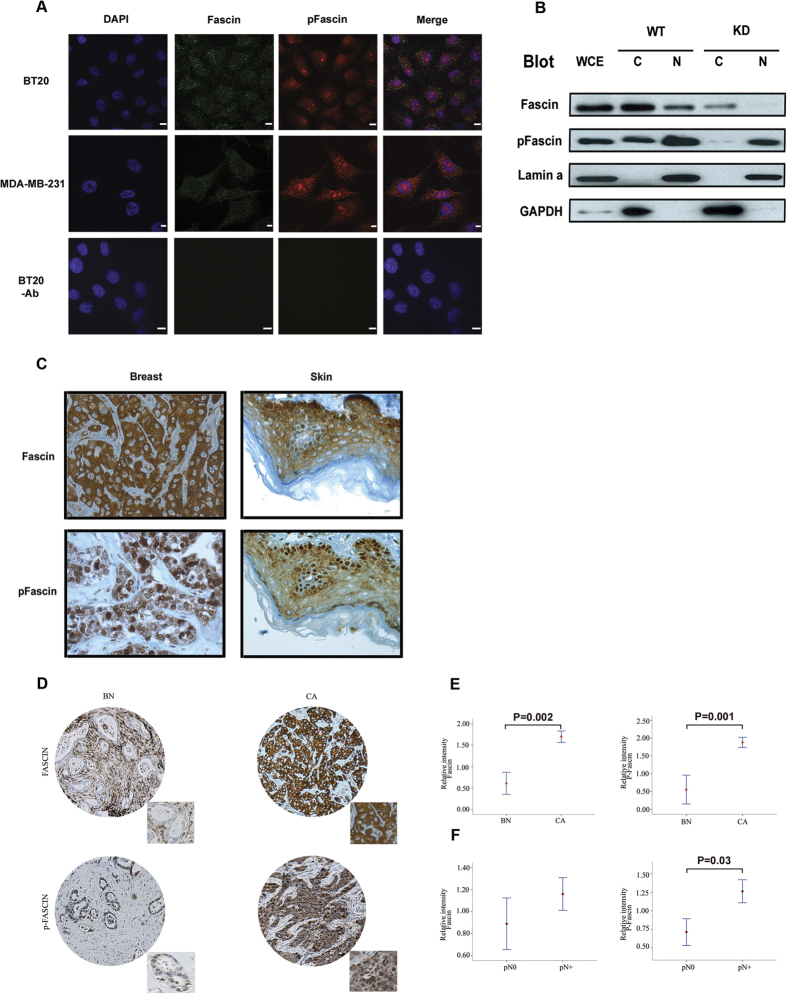
Fascin localizes to the nucleus in both breast cancer cell lines and tissue. Fascin and pFascin show similar localization *in vitro* and *in vivo*. (**A**) BT-20 and MDA-MB-231 cells were grown on coverlips, fixed and stained for confocal microscopy. Primary antibodies against Fascin (green) and pFascin (red) were used followed by visualization using Alexa-conjugated secondary antibodies. Punctate staining corresponding to both Fascin isoforms is visible inside the nucleus. Scale bar, 10 μm. -Ab: primary antibodies omitted. (**B**) Cytoplasmic and nuclear extracts were prepared from WT and Fascin knockdown BT-20 cells and immunoblotted for Fascin and pFascin with corresponding antibodies. GAPDH and Lamin were used as fractionation controls. (**C**) Representative images of immunohistochemical staining of Fascin and pFascin in a multi-tissueTMA. Magnification: 40x. (**D**,**E**) Distribution of Fascin and pFascin in nuclear and cytoplasmic fractions analyzed by immunoblotting. (**D–F**) Fascin and P-Fascin expression across different stages of breast cancer progression. (**D**) Representative images of Fascin and P-Fascin expression in benign tissue (BN) and breast cancer (CA). (**E**) Fascin and P-Fascin protein expression at different stages of breast cancer progression and (**F**) lymph nodes status. Confidence intervals (95%) show normalized mean intensity value units of Fascin and P-Fascin as determined by quantitative evaluation of immunohistochemistry. The y-axis represents numerical values corresponding to the intensity of Fascin and P-Fascin expression (0, negative; 1, weak; 2, moderate and 3, high intensity). pN0: lymph nodes pathologically negative and pN+: lymph nodes pathologically positive.

To investigate the localisation of Fascin to the nuclear compartment, we carried out confocal microscopy coupled to indirect double immuno-staining for Fascin and its phosphorylated form with DAPI marking of the nucleus in two separate breast cancer cell lines (BT-20 and MDA-MB-231). We were able to confirm the presence of both Fascin species inside the nucleus (Fig. 1A), with phosphorylated Fascin (pFascin) yielding a much stronger signal inside the nucleus than total Fascin. This is the first evidence supporting a yet unreported localization of pFascin in the nuclear compartment (Fig. 1A). Interestingly, when signals from both modified and unmodified Fascin were merged, little to no co-localization was detected, suggesting a distinct role for pFascin, in agreement with a recent study by Kelpsch *et al*. which reported very low amounts of fascin in Drosophila nuclei^15^. To quell any possibility of a technical artefact, we isolated cytoplasmic and nuclear extracts from control and Fascin knockdown BT-20 cells and subjected them to western blot analysis (Fig. 1B). The results confirm that Fascin is mainly cytoplasmic whereas the nuclear fraction was enriched with the phosphorylated form. These findings are in line with the data published by Groen *et al*., showing perinuclear and nuclear Fascin staining^14^. To further validate the specificity of our antibodies, we sought to generate *FSCN1* knockout BT20 clones using the Cas9-CRISPR technology. We designed one guide RNA (qRNA) to target exon 1 of the *FSCN1* gene. Six clones were selected for validation. Clones 1 and 5 (C1 and C5) showed loss of protein expression by immunoblotting using the total fascin antibody (Figure S2). More importantly, a reblot of the same nitrocellulose membrane showed that our pFascin antibody was specific to pFascin as the higher molecular weight band disappeared in both C1 and C5 clones (Figure S2).

The next question we asked was whether the observed nuclear localization of Fascin occurred *in vivo*. To this purpose, we interrogated a widespread multi-tissue array from healthy and diseased patients diagnosed with cancer using immunohistochemistry (Fig. 1C). Cores isolated from breast, skin and colon tumor tissues exhibited distinct nuclear and cytosolic staining consistent with the earlier mentioned observations (Fig. 1A).

To provide insights into the potential relevance of nuclear Fascin beyond the constraints of cell lines, we evaluated the expression of nuclear versus cytosolic Fascin in a breast cancer tissue microarray (TMA) from patients with progressive disease. In agreement with our basic studies, pFascin nuclear staining was significantly elevated in carcinomas (CA) compared to benign (BN) (Fig. 1D,E). Mean intensity of total cytosolic Fascin was 0.61 ± 0.33 for BN compared to 1.69 ± 1.03 for CA (p = 0.002), while the mean intensity of nuclear pFascin was highest in CA (1.88 ± 1.11) compared to BN (0.56 ± 0.53) (P = 0.001). Interestingly, a strong correlation was seen when comparing Fascin expression levels and the lymph-nodes status comparing 181 cases with lymph nodes-positive (pN+) and 80 cases with lymph nodes-negative (pN0) (Fig. 1F). The mean intensity levels of Fascin and p-Fascin were higher in pN+ (1.15 ± 0.94 and 1.26 ± 1.10, respectively) compared to PN- cases (0.88 ± 0.34; and 0.72 ± 1.13, respectively). Nuclear p-Fascin expression was found to be significantly associated with lymph nodes positivity (Fig. 1H; P = 0.03) supporting a potential implication in cancer progression.

### Prediction and validation of Fascin NLS

**Figure 2:**
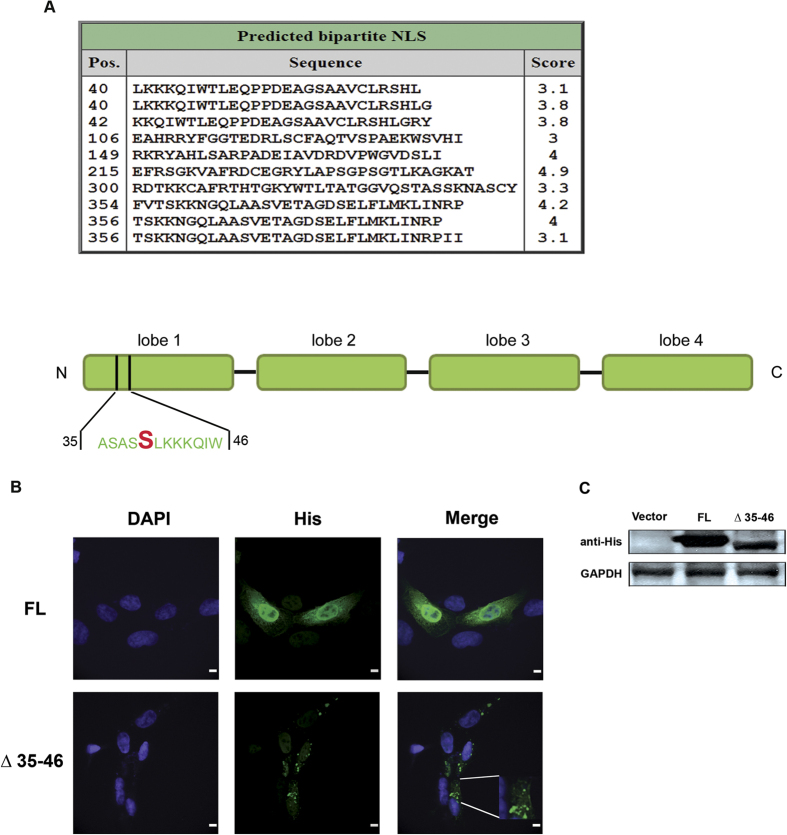
Fascin possesses an intrinsic NLS and amino acids 35–46 spanning the S39 residue are necessary for nuclear translocation. (**A**) Upper panel: screenshot of results from cNLS mapper prediction program for importin α/β-specific NLSs using human Fascin amino acid sequence. Bottom panel: schematic drawing of Fascin deletion mutant used for the subsequent localization experiment by confocal microscopy where amino acids 35 to 46 were removed. In red: amino acid serine 39. NLS scores between 5 ≥ x ≥ 3 confer both nuclear and cytoplasmic localization. (**B**) BT-20 cells were transfected with either full-length or truncated Fascin. Slides were then prepared for confocal microscopy by incubation with an anti-His antibody, followed by visualization using an Alexa-conjugated secondary antibody. Removal of the residues surrounding S39 abolishes Fascin nuclear localization. The enlarged panel shows that the mutant retains its actin binding function Scale bar, 10 μm. (**C**) 293 cells were transfected with FL or mutant Fascin and analyzed by immunoblotting to confirm protein expression levels. FL: full length. The numbers indicate the position of the deleted amino acids.

The presence of a nuclear Fascin implies the presence of an NLS intrinsic to the protein or possibly provided by an interacting partner. To verify the first hypothesis, we analysed the sequence of Fascin through the cNLS Mapper program, which predicts the presence of importin-α dependent NLSs^16^. The algorithm was applied with a cut-off score of 3. NLSs with a score 5 ≥ x ≥ 3 are localized to both nuclear and cytoplasmic compartments according to the authors. Using such criteria, 10 hits were returned. The top three were flanking amino acid serine 39, a major regulatory phosphorylated residue (Fig. 2A). Interestingly, phosphorylation either within or surrounding an NLS is a traditional feature used by cells to control nuclear shuttling^17^,^18^,^19^. Therefore, and based on the predominance of pFascin inside the nucleus, we decided to probe the possible contribution of amino acids surrounding the S39 to nuclear translocation.

To this purpose, we built two His-tagged constructs containing either the full length (FL) Fascin or a deleted version lacking amino acids 35 to 46, which were transfected in BT-20 cells followed by confocal microscopy. In this study, the FL protein localized to both the nucleus and cytoplasm (Fig. 2B), whereas the deletion mutant demonstrated staining reminiscent of perinuclear aggregates while retaining its actin binding capabilities as demonstrated by its localization to the plasma membrane (Fig. 2B). To exclude any effects due to protein instability and degradation, total extracts from transfected cells were analyzed by western blot (Fig. 2C). Although FL Fascin seems to be present in higher quantity, sizeable amounts of the mutant form are still present. Consequently, we can conclude that residues 35 to 46 are primordial for Fascin translocation to the nucleus.

Further supporting the solidity of our finding is the recent discovery we made while searching the GTEX portal database. This database contains RNA expression data within individual tissues coming from multiple human donors using RNA sequencing technology (http://www.gtexportal.org/home/). When looking at exon expression for Fascin, we found that there were predominantly two transcripts that were generated from the *Fscn1* locus. Moreover, the only difference between both transcripts lied in exon 1 where a stretch of 57 nucleotides is absent (Figure S3A). Once translated and aligned, the protein corresponding to the truncated transcript was identical to the full length protein except for a stretch of 19 amino acids. Those 19 amino acids, plus/minus few amino acids, correspond exactly to the NLS identified (Figure S3B). These findings validate our in silico approach and indicate that our truncate construct is realistic and that the cells make an endogenous Fascin which conserves its cytoplasmic capabilities while lacking its nuclear function.

### Fascin interacts with the transcription machinery

**Figure 3:**
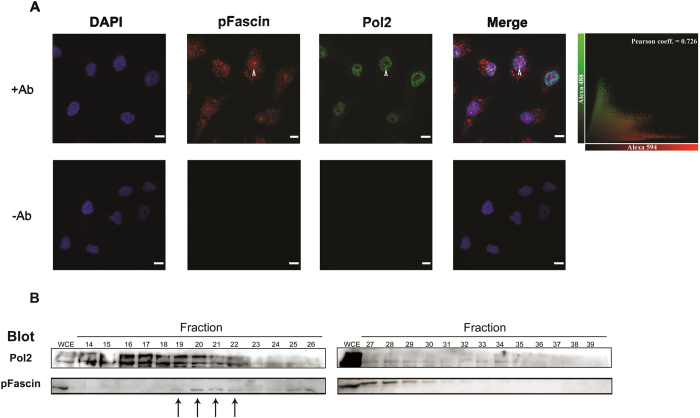
Fascin colocalizes and physically interacts with Pol2 inside the nucleus. (**A**) BT-20 cells were grown on coverlips, fixed and stained for confocal microscopy. Primary antibodies against pFascin (red) and Pol2 (green) were used followed by visualization using Alexa-conjugated secondary antibodies. The arrows point to colocalizing clusters. Top right hand panel: Scatter plot of Fascin and Pol2 intensities. Top right corner: Calculated pearson correlation coefficient. Scale bar, 10 μm. -Ab: primary antibodies omitted. (**B**) BT-20 nuclear protein extracts were fractionated using size exclusion chromatography followed by western blot analysis using the indicated Pol2 and pFascin antibodies. Arrows point to fractions where Pol2 and pFascin co-elute.

The nuclear localization and punctate staining observed for pFascin is similar to a pattern generally displayed by transcription factors^20^,^21^. As such, we tested whether pFascin would exist in a complex with components of the transcriptional machinery. Remarkably, we were able to detect co-localization of pFascin and Pol 2 by confocal microscopy (Fig. 3A). We used the Volocity software to quantify pixel colocalization within the two channels used to detect Pol2 (Alexa Fluor 488) and pFascin (Alexa Fluor 594) for Z-series confocal data^22^. The Volocity-calculated Pearson correlation coefficient of 0.726 indicates a significant degree of 3D colocalization (1.0 = 100% overlap of signal).

Additional evidence for the presence of pFascin within a transcriptional complex containing Pol2 was provided by the use of size exclusion chromatography coupled to western blot analysis. This approach allows the identification of interacting members that might not be seen by co-IP due to the macromolecular nature of these complexes and the possible weakness of the interactions. As expected, we were able to detect the presence of pFascin and Pol2 in the same fractions (Fig. 3B). Taken together, these findings strengthen our belief in the association between pFascin and members of the transcriptional factory.

### Fascin modulates gene expression

**Figure 4:**
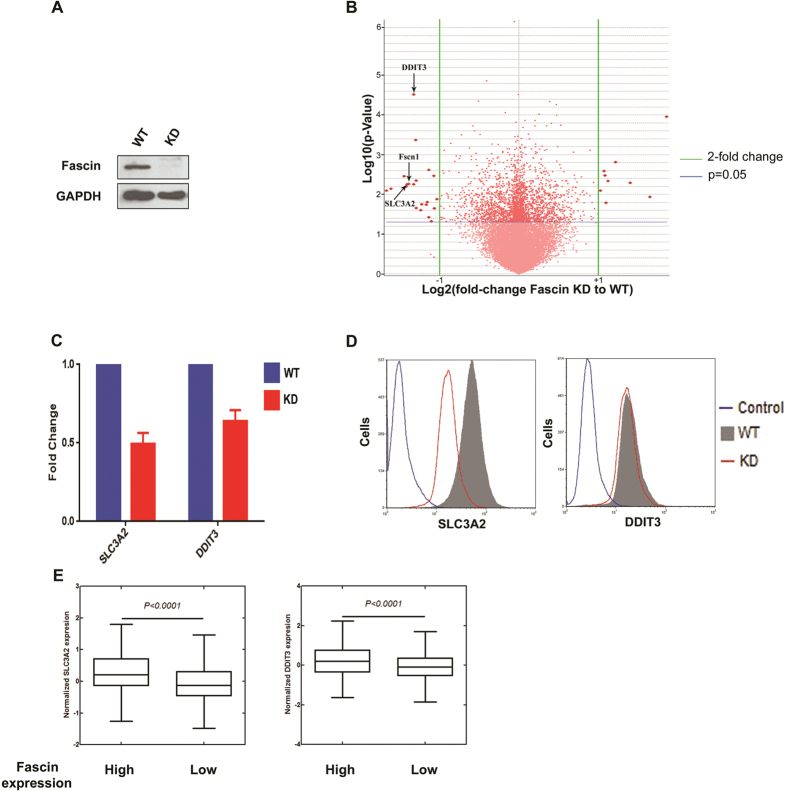
Potential Fascin targets identified by gene expression profiling. (**A**) Western blot of protein extracts from BT-20 WT and stable Fascin knockdown cells showing efficient abrogation of Fascin protein levels. Anti-Fascin (1:1000) antibody was used for fascin detection (upper panel) and anti-GAPDH (1:1000) was used as a loading control (bottom panel). (**B**) Volcano plot analysis of microarray expression data in the Fascin KD and WT cell lines show significant changes in gene expression. For every transcript on the microarray, fold change in expression values of Fascin KD cells compared to WT cells and statistical significance of the observed differences were calculated. Vertical black lines represent a 2-fold variation in gene expression and dots above the horizontal blue line show statistically significant change in gene expression between both cell lines (p ≤ 0.05). (**C**) RT-qPCR data from two targets identified by microarray. mRNA from knockdown and wildtype cells was reverse transcribed into cDNA and analyzed by qRT-PCR. GAPDH was used as an internal control. Error Bars represent SEM from three different expriments. (**D**) The effects of Fascin knockdown on SLC3A2 and DDIT3 protein levels was confirmed by flow cytometry. Anti-DDIT3 (1:200) followed by incubation with the corresponding Alexa-conjugated secondary antibody or FITC-conjugated anti-SLC3A2 were used for each target. Mean fluorescence intensity (MFI) is indicated on the abscissa. (**E**) Box plots showing the differential expression between Fascin High and Low tumors (TCGA data) of SLC3A2 and DDIT3. The two-tailed t-test P value is shown.

To investigate the impact of Fascin on the regulation of gene expression, we analyzed RNA isolated from control and stable Fascin knockdown BT-20 cells by microarray analysis (Fig. 4A). The goal was to identify genes that were under direct control of Fascin. Compilation of the data from 3 biological replicates with at a least a statistically relevant 2-fold change yielded a significant, yet small cohort of target genes (Fig. 4B). Of the validated genes, two selected representative genes, *DDIT3* (DNA-damage-inducible transcript 3) and *SLC3A2* (a plasma membrane transporter that regulates intracellular amino acid levels), formerly shown to be implicated in mediating cancer progression^23^,^24^, showed striking decrease in mRNA levels upon Fascin knockdown (Fig. 4C). The same trend was observed when protein levels for both genes were assayed by flow cytometry even though the extent of the reduction was more pronounced for SLC3A2, as demonstrated by the marked shift in mean fluorescence intensity (MFI) in the knockdown cells (Fig. 4D). To further confirm this association, we interrogated the TCGA database for *SLC3A2* and *DDIT3* levels in Fascin high and low tumor samples (Fig. 4E). As expected, we found that *SLC3A2* and *DDIT3* levels positively and significantly correlated with Fascin levels further supporting a transcriptional role for Fascin (Fig. 4E).

### Fascin localizes to chromatin and influences gene transcription

**Figure 5:**
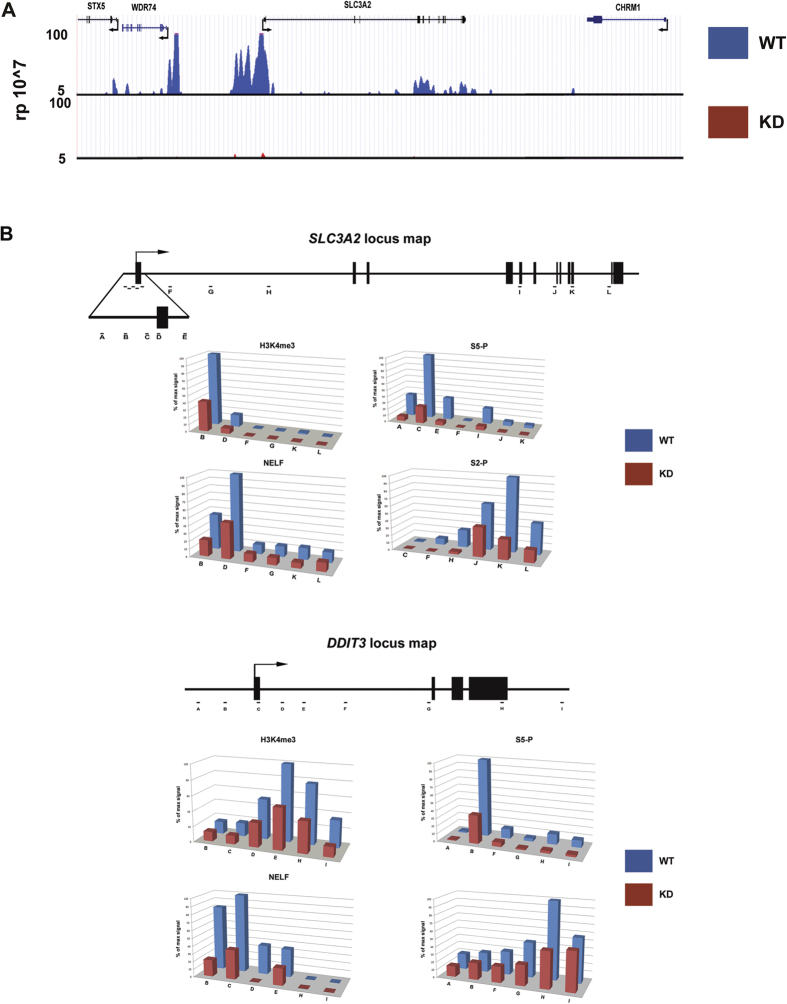
pFascin localizes to chromatin and positively regulates Pol2-mediated transcription processes. (**A**) pFascin ChIP-Seq enrichment profiles for WT (upper panel) and KD (bottom panel) BT-20 cells on the SLC3A2 locus. (**B**) pFascin positively regulates transcription on the SLC3A2 and DDIT3 locus. ChIP analysis of phosphorylated Pol2 using antibodies against phosphorylated ser-5 and ser-2 reveal sizable loss of of both residues. H3K4me3 and NELF levels on SLC3A2 and DDIT3 mirror those of phosphorylated Pol2. 2 PCR replicates: 6, ChIP replicates: 2.

As shown above, microarray gene profiling using mRNA from wild type and Fascin-knockdown BT-20 cells allowed for the identification of gene candidates under the transcriptional control of Fascin. To confirm that Fascin-mediated gene transcription is a result of a direct action of Fascin at the chromatin level, we immunoprecipitated chromatin from control and Fascin-knockdown cells using a pFascin specific antibody coupled with high-throughput sequencing (ChIP-seq) to map the regions possibly bound by pFascin. Interestingly, we were able to detect specific regions bound by pFascin with at least 15% of the total peak count found in the promoter region (Table S2). Of relevance, GO term analysis revealed enrichment in pathways involved in cancer (Table S1). More importantly, pFascin was enriched at the *SLC3A2* gene promoter, suggesting a possible role in transcription initiation (Fig. 5A).

To further understand the connection between Fascin and transcription we performed ChIP for the H3K4me3 mark, Pol2 phosphorylated on serine 5 (S5-P, initiating Pol2), serine 2 (S2-P, elongating Pol2) and NELF (establishes Pol2 pausing) at both target genes. Strikingly, we observed loss of H3K4me3 from Fascin targets upon Fascin knockdown (Fig. 5B). Previous reports indicate that H3K4me3 plays a pivotal role in Pol2 recruitment^25^,^26^. Therefore, our own data showing diminished H3K4me3 at Fascin target genes in knockdown cells, plus our data indicating an interaction between Pol2 and Fascin suggest that Pol2 recruitment may be impaired in Fascin knockdown cells. Supporting this premise, we also saw a loss of phosphorylated Pol2 and the Pol2-associated factor NELF at these genes (Fig. 5B). Our results support the presence of pFascin at the promoter where it is involved in establishing a mature pre-initiation complex. Thus, the observed decreases in transcript levels of Fascin targets genes in Fascin-knockdown cells would reflect the disruption of these transcriptional events (Fig. 4C).

### Fascin colocalizes with H3K4me3 and interacts with RbBP5

**Figure 6:**
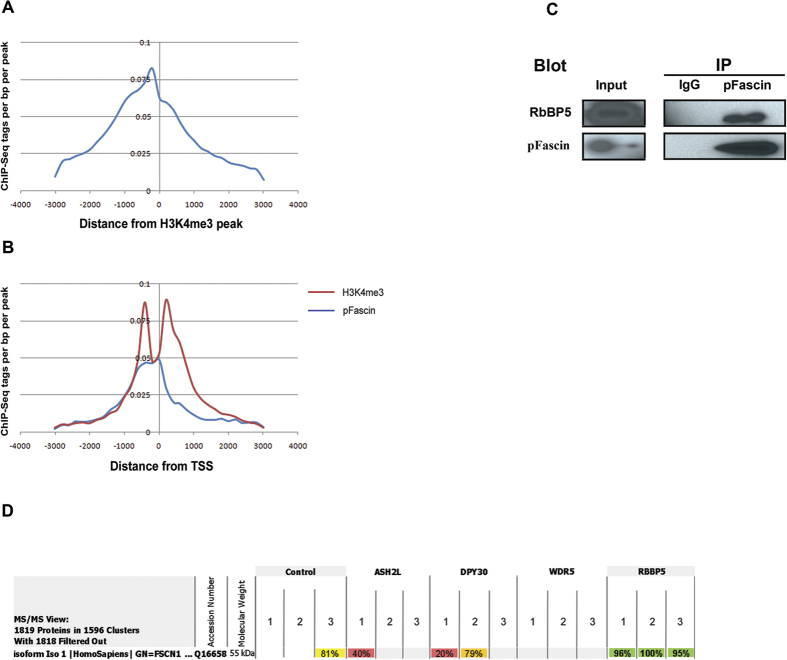
ChIP-seq analysis reveals remarkable colocalization of pFascin with H3K4me3. (**A**) Histogram representing pFascin binding sites distribution and density relative to H3K4me3 peaks. (**B**) Histogram representing pFascin and H3K4me3 binding sites distribution and density relative to the transcription start site (TSS). (**C**) The interaction of Fascin protein with H3K4 methylatransferase complex was analyzed following immunoprecipitation of BT-20 extracts with antibodies directed against pFascin followed by western blot analysis and immunochemical detection of RbBP5 and pFascin. (**D**) Screenshot from Scaffold 4 program showing the results of mass spectrometry data of H3K4me3 core subunits interactors. Fascin is clearly and specifically interacting with RbBP5. For each subunit, the immunoprecipitation was done in triplicate. The percentages represent the peptide identification probability.

Recent studies dissected the role of H3K4me3 in gene activation by transcription factors and in the assembly of the pre-initiation complex^25^,^26^. Therefore and in consideration of our results, we hypothesized that pFascin might regulate transcription through modulation of H3K4me3 complex recruitment. First, we analyzed pFascin occupancy in comparison to previously published H3K4me3 ChIP-seq data. We observed that both profiles almost overlapped with pFascin being slightly upstream hinting to a role in the methylation of the downstream nucleosomes at the promoter (Fig. 6A). Moreover, the absence of pFascin downstream of the transcription start site (TSS) supports a role in the initiation of transcription (Fig. 6B). Since these results pointed toward a possible relation to H3K4 methylation, we investigated the possibility of pFascin as being a subunit of the HMT complex. This complex is mainly composed of four core subunits (one of which is RbBP5) and of enzymatic subunits from the Set1 or MLL family^27^. To this purpose, we performed co-immunoprecipitation of pFascin from nuclei of BT-20 cells followed by western blot analysis for the detection of RbBP5. As hypothesized, we indeed detected a specific interaction between pFascin and RbBP5, which strongly indicating that pFascin could be a member of the H3K4 methyltransferase complex (Fig. 6C). To further confirm our findings, we analyzed raw mass spectrometry data from a study by Van Nuland *et al*.^28^ where the authors immunoprecipitated the different core components of the H3K4 methyltransferase complex and then subjected the eluates to mass spectrometry to identify their binding partners. As expected, we were able to specifically detect an interaction between RbBP5 and Fascin (Fig. 6D).

### Fascin regulation of SLC3A2 affects mTOR pathway

As noted above, *SLC3A2* was identified as a Fascin-targeted gene. Cancer cells have increased metabolic demands necessitating a heightened uptake of glucose and amino acids from the extracellular environment. One of the major components of the amino acid uptake machinery is SLC3A2. It plays the role of a chaperone for several amino acid transporters where it facilitates their trafficking to the plasma membrane^29^,^30^. One of SLC3A2 binding partners is SLC7A5. Once formed, this heterodimer mediates the import of extracellular leucine in exchange of intracellular glutamine through its antiport activity^31^. Several studies showed that this action is responsible for amino acid mediated activation of mTORC1 and of its downstream signalling affecting cell growth and survival^32^,^33^.

**Figure 7:**
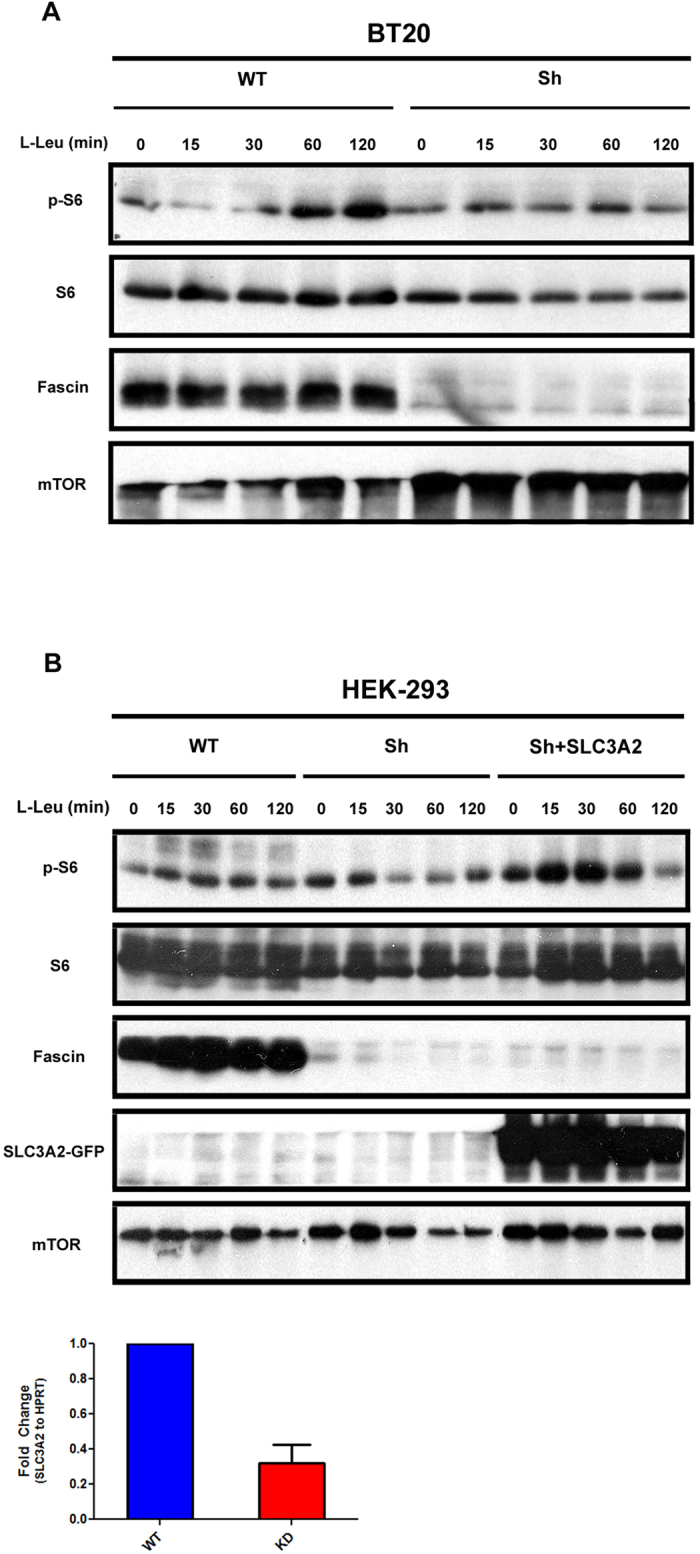
Fascin mediated SLC3A2 expression modulates mTORC1 activation by Leucine. (**A**) Time course of mTORC1 activation by Leucine in serum and amino acid starved WT and Fascin KD BT-20 cells visualized by S6 phosphorylation. (**B**) (Top) WT, Fascin knockdown (sh) and Fascin knockdown reconstituted with GFP-tagged SLC3A2 HEK-293 cells were serum and amino acid starved then stimulated with Leu over a time course. mTORC1 activation was visualized by S6 phosphorylation. (Down) RT-qPCR data for *SLC3A2*. mRNA from Fascin knockdown and wildtype HEK-293 cells was reverse transcribed into cDNA and analyzed by qRT-PCR. HPRT was used as an internal control. Error Bars represent SEM from three different experiments.

We investigated the capacity of leucine to activate mTOR as a reporter for SLC3A2 regulation by Fascin since a decrease in the transporter levels should equate to a decrease in mTORC1 activation due to lower levels of amino acids, namely leucine^32^,^34^. To this purpose, serum and amino acid-starved BT-20 cells expressing WT or Fascin KD were loaded with glutamine then stimulated over a time course with leucine followed by monitoring of mTORC1 activity through S6 phosphorylation (Fig. 7A). As expected, WT cells responded gradually to leucine stimulation however Fascin KD cells were unable to trigger S6 phosphorylation (Fig. 7A). To exclude that this observation was artefactual, we generated HEK-293 cells where Fascin was knockdown followed by transient reconstitution with GFP-tagged SLC3A2 and mTORC1 activation was monitored as described above. As in Fig. 7A, HEK-293 cells with reduced Fascin expression didn’t respond to leucine stimulation as visualized by lack of S6 phosphorylation (Fig. 7B). However, transient reconstitution with SLC3A2 allowed for recovery of mTORC1 activation (Fig. 7B). Of note, HEK293 cells had also reduced SLC3A2 transcript levels when Fascin was knock down using shRNA (Fig. 7B). We would like to mention that we used HEK-293 cells due to a better transfection efficiency rate compared to BT-20 cells. In summary, this finding confirms that the pFascin-SLC3A2 regulatory axis is functional and supports the idea that pFascin modulation of gene expression has relevance to cell metabolism.

## Discussion

Fascin has been extensively investigated in relation to its actin binding and bundling function that take place in the cytosol. In this study, we established a novel nuclear function of Fascin in transcriptional regulation via its phosphorylated form. We identified pFascin to be primarily localized to the nucleus in several cell types. Bioinformatic and mutational analyses revealed a putative NLS flanking the regulatory S39 residue of Fascin. We believe that this proximity is not coincidental as phosphorylation near an NLS has previously been reported as one of the regulatory mechanisms of nuclear import^33^. For example, Hubner *et al*. were able to show that phosphorylation of serines located in close proximity to the SV40 large T-antigen NLS increased its nuclear import in a rat hepatoma cell line^35^,^36^. In agreement with our findings, a recent study by Jayo *et al*. revealed Fascin as a perinuclear protein, where the non-phosphorylatable Fascin (S39A) was unable to translocate to the perinuclear space as it was constitutively bound to actin^37^. The non-canonical presence of pFascin inside the nucleus is analogous with previous findings on other cytoskeletal proteins, including actin and actin-binding proteins, which were involved in nuclear processes and acting as members of the transcription machinery and/or as partners of RNA polymerases^38^,^39^,^40^. In particular, nuclear actin has been shown to exert its effects on gene expression through (i) binding of the three RNA polymerases; (ii) regulation of transcript processing by snRNP binding; (iii) control of mRNA nuclear export through hnRNP binding; and (iv) modulation of chromatin states by binding chromatin-remodeling and histone acetyltransferase (HAT) complexes^41^,^42^,^43^,^44^,^45^,^46^. Other than actin, a handful of actin binding proteins (ABP) such as Filamin A, Gelsolin, the ARP family have been shown to translocate to the nucleus where they affect several arms of the transcriptional processes involved in gene expression^38^,^39^,^40^. Our study reveals a novel nuclear function for Fascin mediated through interaction of pFascin with Pol2, a prominent regulator of transcription. This interaction impacted gene regulation as we identified several targeted genes differentially expressed when levels of Fascin were manipulated.

The interaction of nuclear Fascin with the transcriptional machinery is significant given the broad implication of this dynamic and multifaceted process to gene regulation. Transcription is tightly regulated at several stages, one of which is at the level of the chromatin. The chromatin is organized into nucleosomes, which are made of histone octamers. Under the action of several ATP-dependent enzymes, these histones can undergo dynamic changes and post-translational modifications influencing the physical state of the chromatin^47^. These modifications serve as docking modules for chromatin readers, many of which are primordial for transcription^48^. One of the most studied modifications is H3K4me3. It is conserved at active promoters from yeast to humans and is recognized as tightly correlated with Pol2-mediated transcription through the regulation of pre-initiation complex assembly^25^,^49^. In mammalians several complexes with H3K4 methylation capabilities have been identified with proteins belonging to the mixed-lineage leukemia (MLL) and hSet1A/B family being the most studied. All complexes are made of core, enzymatic and complex-specific subunits, which can be shared or are unique to each^27^,^50^. The core consists of ASH2L, DPY30, RBBP5 and WDR5 proteins, which are shared across all complexes. Several complex specific proteins, such as menin and WDR82, have also been identified and are believed to be essential for recruitment of the complexes to specific genomic loci^51^,^52^.

**Figure 8:**
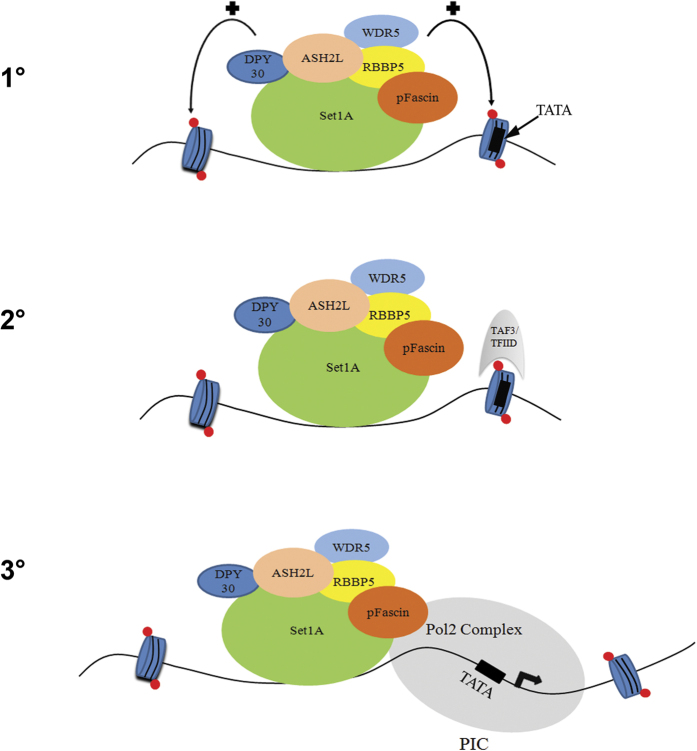
Hypothetical Model for pFascin Interaction with HMT Complex and to Promote PIC Assembly. Our findings suggest a model where, at target genes, (1) pFascin promotes H3K4me3 through interactions with RbBP5 which in turn (2) modulates TFIID recruitment through direct interaction between H3K4me3 and TAF3 which finally (3) promotes the assembly of PIC. Red dots: H3K4me3.

In our study, genome-wide localization of pFascin showed co-occurrence with the H3K4me3 mark, and further ChIP analysis revealed the positive role of Fascin on histone methylation and pol2-mediated transcription at specific target genes. The distribution of pFascin around the TSS suggests that it may contribute to H3K4me3 presence in downstream nucleosomes. This profile is reminiscent of that of histone methyltransferase complexes such as ASH2L, where binding precedes that of this histone mark^53^. Strengthening a role for Fascin in H3K4 methylation, we identified herein a novel interaction between pFascin and the RbBP5 core subunit of the H3K4 HMT through pFascin co-immunoprecipitation. These findings were further confirmed by a mass spectrometry analysis of the H3K4 methyltransferase complex interactome in which Fascin was identified as a specifically interacting partner of RbBP5^28^. Based on our findings, we propose a novel model for pFascin in the regulation of gene expression through H3K4me3 modulation (Fig. 8). In this model, pFascin promotes H3K4me3 at specific promoters through interaction with RbBP5 and its interacting partners to participate to the recruitment of TFIID followed by formation of the pre-initiation complex with Pol2. We believe that in this model, pFascin role is target-specific as evidenced by the ChIP-seq data, where pFascin was selectively present at approximately 15% of all genes within the human genome. One can hypothesize that pFascin possesses targeting capabilities on the HMT complex redirecting it to genes important for cancer. Such phenomena, where subunits of the H3K4 methylation complex play a role in gene targeting, have been observed for example with Menin where it directs the MLL histone methyltransferase complex to the *HOX* gene locus^52^,^54^.

Our group and others have previously demonstrated the impact of Fascin on cancer progression and invasion with regards to its role in the reorganization of the actin cytoskeleton^8^,^11^. As mentioned earlier, we believe that the multitude of cellular processes that Fascin is implicated in cannot solely be attributed to its cytoplasmic role. Advocating for our hypothesis, one of the newly identified Fascin target gene, SLC3A2, has been shown by several groups to modulate mTORC1 activation through its amino acid transporter function^32^,^34^,^55^. A recent study showed that loss of SLC3A2 leads to major imbalances in amino acid intracellular levels and more specifically a decrease in leucine concentration by 2 fold^56^. Our work shows that, in Fascin KD cells, mTOR is less responsive to leucine stimulation. We believe that this phenotype is due to lower import of leucine in exchange for glutamine under the action of the SLC7A5/SLC3A2 antiporter. Glutamine, on its own, has also been shown to activate mTORC1^32^,^57^. However, we exclude any confounding effect from glutamine as de la Ballina *et al*. showed that SLC3A2 loss leads to accumulation of glutamine inside the cell^56^. Moreover, we designed our experiment to possibly account for such variability with preloading of the cells with glutamine prior to treatment with leucine. Hence, in addition to elucidating the role of pFascin impact on transcription, we demonstrated that changes to SLC3A2 levels have the potential to affect cell growth and survival through modulation of leucine-mediated mTOR activation. Together, our results documenting a novel function of pFascin in transcriptional regulation with impact on cell metabolism open up new research avenues with implication in physiological and pathological contexts.

## Experimental Procedures

### Cell lines and cell culture

BT-20, MDA-MB 231 and HEK-293 cell lines were obtained from American Type Culture Collection and maintained in RPMI 1640 (Hyclone) supplemented with 10% FBS and supplemented with penicillin and streptomycin antibiotics. Fascin knockdown BT-20 cells were generated in a polyclonal population. Target sequences were cloned into the pSUPER.retro.puro vector system and transfected in Phoenix retroviral expression packaging cell line using Genejuice transfection reagent (Novagen). Following transfection, Phoenix cells were incubated at 37 °C in an atmosphere of 5% CO_2_ for 48 hrs and were then selected for efficiency of transfection using culture medium supplemented with puromycin. After complete selection, Phoenix cell viral supernatant containing polybrene (1,5-dimethyl-1,5-diazaundecamethylene polymethobromide, hexadimethrine bromide, Sigma Aldrich) was used to infect BT-20 target cells. The cells were then incubated with the viral supernatant for 48 hrs at 37 °C in an atmosphere of 5% CO_2_. Cells stably transfected were selected using culture media supplemented with 1 μg/ml puromycin. Target sequence used for the stable knockdown of Fascin is: 5′-GCCTGAAGAAGAAGCAGAT-3′.

To generate the fascin knockout using CRISPR-Cas9 system, sequences of human codon optimized Cas9 and guide RNAs (gRNAs) targeting the *Fscn1* gene were obtained from Mali *et al*. (sequence: GCGCTGCCCGCCTCGTCAGGGGG, position: chr7:5632718-5632740)^58^. The gRNA plasmid was synthesized by IDT. A plasmid encoding for Cas9, the gRNA and a plasmid encoding for GFP were co-transfected into BT-20 cells using Lipofectamine 3000 (Invitrogen) as per manufacturer’s instructions. 48 h post-transfection, high GFP-expressing cells were sorted into 96-well plates as single clones. Colonies were serially transferred to larger culture vessels until enough cells were available for *Fscn1* gene disruption screening by immunoblotting.

Fascin knockdown HEK-293 cells were generated in a polyclonal population. The target sequences cloned into the pLKO.1 vector system was purchased from Sigma Aldrich (Clone TRCN0000289001) and transfected in 293-T cells with corresponding lentiviral packaging and envelope plasmids. Following transfection, 293-T were incubated at 37 °C in 5% CO_2_ for 48 hrs and viral supernatant containing polybrene was used to infect HEK-293 target cells. The cells were then incubated with the viral supernatant for 48 hrs at 37 °C in 5% CO_2_. Cells stably transfected were selected using culture media supplemented with 1 μg/ml puromycin.

For mTORC1 stimulation, amino acid starvation was performed by depriving cells of FBS for 20 hr and then incubation with D-PBS containing 0.90 mM CaCL_2_, 0.45 mM MgCl_2_, 15 mg/L phenol red, 20 mM HEPES and 1 g/L D-glucose (starve medium) for 3 hr. Amino acid starved cells were then cultured in the presence of 10 mM L-glutamine for 1 hr at 37 °C. The medium was then removed and cell monolayers washed twice with starve medium before adding the starvation medium supplemented with leucine (no glutamine) at the concentration found in RPMI (50mg/L) for the indicated times.

### Plasmid construction and expression

Full length (FL) Fascin constructs were cloned in the pCDNA 3.1/HisA vector. The forward primer sequence 5′-CGGAATTCAGACCGCCAACGGCACAGCC-3′ and the reverse primer sequence 5′-GCTCTAGACTAGTACTCCCAGAGCGA-3′ were used to insert wild-type Fascin 1 between the EcoRI and XbaI sites. A Fascin 1 deletion mutant lacking the amino acids 35 to 46 (consequently the phospho-S39 residue) was generated by overlap extension PCR as described previously (Lee J. *et al*., 2010) using the above mentioned primers, the forward primer sequence 5′-GCGTTCGGGTTCAAGGTGAACACGCTGGAGCAGCCCCCTGAC-3′ and the reverse primer sequence 5′-GTCAGGGGGCTGCTCCAGCGTGTTCACCTTGAACCCGAACGC-3′ which yields a PCR product deleted for the aforementioned amino acids. The generated proteins contain a polyhistidine tag at the N-terminus. Constructs were validated by sequencing and western blot analysis. Both plasmids were transfected in BT-20 cells grown on glass coverslips in 6-well plates according to manufacturer’s instructions using 2.5 μg of plasmid DNA. Cells were then prepared for confocal microscopy as described using an antibody against histidine (Santa Cruz, sc-803, 1:200).

Full length wild type SLC3A2 was cloned in the pEGFP-C1 vector. The forward primer sequence 5′-CCCAAGCTTCGGAGCTACAGCCTCCTGAAG-3′ and the reverse primer sequence 5′-CGGGATCCTTAGGCCGCGTAGGGGAAG-3′ were used to insert wild-type SLC3A2 between the HindIII and BamHI sites respectively. The generated protein contains a FLAG tag at the C-terminus. All constructs were validated by sequencing and western blot analysis.

### Cytoplasmic/nuclear fraction isolation

Cell monolayers in 15 cm dishes were washed twice with cold PBS and scraped with 500 μl of cold buffer A (10 mM HEPES, 1.5 mM MgCl2, 10 mM KCl, 0.5 mM DTT, 0.05% NP40, 1 mM PMSF, 2 mM Na3VO4 and protease inhibitor mixture) and left on ice for 10 min. The extracts were then centrifuged at 3000 rpm for 10 min at 4 °C. The supernatant was kept and labelled as cytoplasmic fraction. The remaining nuclei were extracted in 200 μl of buffer B (5 mM HEPES, 1.5 mM MgCl2, 0.2 mM EDTA, 0.5 mM DTT, 26% glycerol, 1 mM PMSF, 2 mM Na3VO4, 10 mM NEM, 420 mM NaCl and protease inhibitor mixture). Pellets were homogenized with a probe sonicator. The suspension was then centrifuged at 14,000rpm for 30min at 4 °C. The supernatant was labelled as the nuclear fraction.

### Confocal microscopy

Cell monolayers grown on glass coverslips in 6-well plates were washed twice with PBS and fixed at room temperature (RT) in 1% PFA containing 0.25% Triton X-100 for 15 min. The cells were then washed twice with PBS and blocked in blocking buffer (PBS, 1% BSA, 2% normal goat serum) for 30min. Antibodies targeting Fascin (Santa cruz, sc-21743, 1:200), pFascin (ECM biosciences, FP-2661, 1:1000), Pol2 (Covance, 8WG16, 1:200), mTOR (Cell signalling technologies, 2983, 1:500) were then added directly to the blocking buffer for 1 hour. The cells were then washed three times in PBS and incubated in blocking buffer containing respective Alexa-conjugated secondary antibodies (Invitrogen, 1:1000) and incubated for 1 hour at RT. The cells were then washed three times in PBS and mounted on slides with vectashield containing DAPI. Images were captured using a 40x or 60x oil immersion objective on the Wave FX spinning disk confocal microscopy system (Quorum technologies) and analyzed using Volocity.

### Tissue microarray (TMA) and immunohistochemistry (IHC)

The use of human tissues for this study was approved by the Alberta Cancer Research Ethics Committee (ACREC) and carried out in accordance with the approved guidelines. Tissue cores from a cohort of 243 patients, being 9 fibroadenomas and 234 carcinomas diagnosed with progressive breast cancer disease were assigned a diagnosis by a certified pathologist (Table S3). After obtaining informed consent from each patient, a tissue microarray (TMA) was constructed using a manual tissue arrayer (Beecher Instruments, Silver Spring, MD) and immunohistochemistry performed as we previously described (Xu *et al*., 2010). Briefly, the reaction was carried out using the NexESimmunostainer (Ventana Medical Systems, Tuscon, AZ) on 4 μm silane-coated slides (Sigma, St. Louis, MO), dried overnight at 37 °C and dewaxed, rehydrated, and boiled (microwave) in citrate buffer (pH 6.0) for antigen retrieval. Incubations with the primary antibodies diluted in PBS were conducted for: anti-Fascin (Santa Cruz, sc-21743, 1:100) and anti-pFascin (ECM biosciences, FP-2661, 1:50). Secondary antibody was an indirect biotin-avidin system with a universal biotinylated immunoglobulin. Positive controls (a tissue known to contain the antigen under study) were included in all reactions in accordance with manufacturer´s protocols. Negative controls were obtained by omitting the specific primary antibodies.

Slides were analyzed blindly to the clinical aspects and conducted by two independent certified pathologists. Each core was scanned in low power field to choose the most stained area predominant in at least 10% of tumor cells^59^. Protein expression was assessed using a four-tiered system (0, negative; 1, weak; 2, moderate; and 3, high expression). Each core was evaluated separately and a final score for each case was achieved by averaging the total intensity value of all cores within a specific patient sample (Xu *et al*., 2010). For statistical analysis, samples were categorized into two groups: (N) negative/weak and (P) moderate/strong positive cases. For frequency analysis in contingency tables, statistical analyses of associations between variables were performed by the Fisher’s exact test and for continuous variables the non-parametric Mann–Whitney U test. A p-value < 0.05 was considered significant.

### RNA isolation and microarray analysis

BT-20 control or Fascin knockdown cells were grown to 80% confluence. Total RNA was then isolated using the RNeasy extraction kit (Qiagen). First strand cDNA synthesis was performed using Superscript II reverse transcriptase (Invitrogen) and submitted for microarray analysis to Genome Quebec. The raw data was imported and analyzed using the FlexArray software package (McGill University and Genome Quebec Innovation Centre). Differentially expressed genes were characterized as having a fold-change ≥2 relative to the control cells with a p-value < 0.05.

### Co-immunoprecipitation

Equal amounts of protein from nuclear extracts were incubated with anti-Fascin or anti-pFascin antibodies overnight at 4 °C (Fascin: Santa cruz, sc-21743; pFascin: ECM biosciences, FP-2661). The immune complexes were precipitated with protein G magnetic beads (Millipore) in RIPA buffer containing 1 mM PMSF, 2 mM Na3VO4, 10 mM of NEM and protease inhibitors (Roche) for 30min at 4 °C and samples were analyzed by Western blot analysis with indicated antibodies. As a negative control, we used protein extracts incubated with whole serum IgG (Sigma Aldrich, I8140 and I5381).

### Fast protein liquid chromatography (FPLC)

If two proteins are found in the same complex, they are assumed to co-elute in the size-excluding columns. Fast protein liquid chromatography (FPLC) ÄKTA purifier 10/100 system was used to co-elute interacting proteins. Nuclear extracts of BT20 cells were loaded onto superoseTM 6 column, degased and pumped at flow rate of 0.25 ml/min. All eluents were collected at 0.5 ml/fractions. For western blot analysis, 200 μl of each fraction was loaded to 10% SDS-PAGE gel followed by western blot analysis as described below.

### SDS PAGE and western blot analysis

Equal amounts of protein or equal volumes of elution fraction (FPLC) were subjected to electrophoresis through 10% SDS-PAGE gels and transferred to PVDF membranes (Millipore). Membranes were blocked overnight at 4 °C in PBS containing 5% dried milk then probed overnight at 4° with the indicated primary antibodies (Fascin, Santa cruz, sc-21743, 1:1000; pFascin, ECM biosciences, FP-2661, 1:1000; Flag, Sigma-Aldrich, F1804; Pol2, Santa-cruz, sc-899, 1:1000; RbBP5, Bethyl Laboratories, A300-109A, 1:1000; Set1A, Bethyl Laboratories, A300-289A, 1:1000; mTOR, cell signalling technologies, 2983, 1:1000; S6, cell signalling technologies, 2217, 1:1000; pS6, cell signalling technologies, 2215, 1:1000). Membranes were then washed three times with TBST and incubated for 1 h with secondary antibody coupled to HRP (Millipore, 1:10000) followed by 3 washes in TBST and then chemiluminescent detection (ECL, Millipore).

### Real Time RT-PCR

Total RNA was isolated from control and knockdown BT-20 cells using the RNAeasy kit following the manufacturer’s instructions (Qiagen). The RT reaction was accomplished with qScript cDNA mix (Quanta biosciences) according to the manufacturer’s instructions using 1ug of RNA in 20 μl final volume. Primers were designed against target genes using Primer-BLAST 3.0 (NCBI). The cDNA was diluted 5 times in nuclease free water and PCR reactions containing 1 × SYBR Green Mix (Promega), 2 μl of cDNA, and 100 nM primers were prepared in 96- well plates read in an Applied Biosystems 7500 fast Real-time PCR instrument (Applied Biosystems). Values were normalized to those of GAPDH.

### ChIP assay

The ChIP experiment was done as described using control and Fascin knockdown BT-20 cells with a few modifications^60^. For immunoprecipitation, 1 mg of protein extract was pre-cleared for 1 h with 20 μL of protein G magnetic beads slurry (Millipore) before addition of indicated antibodies. Two micrograms to 4 μg of each antibody was added to the samples and incubated overnight at 4 °C in the presence of 20 μL of protein G beads pre-blocked with 1 mg/mL BSA. When antibodies against phosphorylated Pol2 CTD were used, immunocomplexes were precipitated using anti-mouse IgM/protein G-Sepharose beads (Sigma-Aldrich). DNA was purified and resuspended in 100 μl of water for subsequent qPCR reactions. Primers against multiple regions of *DDIT3* and *SLC3A2* locus were designed with Primer Blast^61^. PCR products range in size between 70 and 150 bp. Ten-microliter PCR reactions containing 2 × SYBR Green Mix (Promega), 2 μl of the ChIP-enriched DNA, and 100 nM primers were set up in 96-well plates. Plates were read in an Applied Biosystems 7500 Fast Real-time PCR instrument (Applied Biosystems). To calculate enrichment for the ChIP samples, beads alone ChIP material (no antibody) was used to subtract and input DNA values were used to normalize the values.

### ChIP-seq and bioinformatics analysis

All ChIP-seq data sets were aligned using BWA (version 0.7.4) to the human reference genome (GRCh37/hg19). Only the sequences with MAPQ score above 20 were kept for further analysis. The alignment files were then analyzed with MACS2 v. 2.0.10 using a 0.001 q-value cutoff^62^ and using KD Fascin alignment as reference to identify the pFascin binding peaks. The peaks identified by MACS2 were annotated using HOMER^63^ to obtain pFascin peaks distribution in the human genome. HOMER was also used to generate the bedgraph file to visualize the ChIP-seq results in the UCSC Genome Browser.

### Mass spectrometry data analysis

The mass spectrometry data, identifier PXD000172, generated by Van Nuland *et al*. was downloaded from the Proteome Xchange Consortium database (http://proteomecentral.proteomexchange.org)^28^. All MS/MS samples were reanalyzed using Mascot (Matrix Science, London, UK; version 2.4.1). Mascot was set up to search the nx_b201205 database assuming the digestion enzyme trypsin. Mascot was searched with a fragment ion mass tolerance of 0.50 Da. Scaffold (version Scaffold_4.2.1, Proteome Software Inc., Portland, OR) was used to validate MS/MS based peptide and protein identifications. Peptide identifications were accepted if they could be established at greater than 95.0% probability by the Peptide Prophet algorithm^64^ with Scaffold delta-mass correction. Protein identifications were accepted if they could be established at greater than 99.0% probability and contained at least 2 identified peptides. Protein probabilities were assigned by the Protein Prophet algorithm^65^. Proteins that contained similar peptides and could not be differentiated based on MS/MS analysis alone were grouped to satisfy the principles of parsimony. Proteins sharing significant peptide evidence were grouped into clusters. Proteins were annotated with GO terms from NCBI^66^.

## Additional Information

**How to cite this article**: Saad, A. *et al*. Insights into a novel nuclear function for Fascin in the regulation of the amino-acid transporter SLC3A2. *Sci. Rep.* **6**, 36699; doi: 10.1038/srep36699 (2016).

**Publisher’s note**: Springer Nature remains neutral with regard to jurisdictional claims in published maps and institutional affiliations.

## Supplementary Material

Supplementary Information
