# Supplementary material for: Insights into a novel nuclear function for Fascin in the regulation of the amino-acid transporter SLC3A2
Source: Sci Rep. 2016 Nov 7;6:36699. doi: 10.1038/srep36699 (PMC5098188; doi:10.1038/srep36699)
Supplement: Supplementary Information [file srep36699-s1.pdf]

Supplemental Data

Insights into a novel nuclear function for Fascin in the regulation of the amino-acid transporter SLC3A2

Amine Saad, Krikor Bijian, Dinghong Qiu, Sabrina Daniela da Silva, Maud Marques, Chia-Hao Chang, Hassan Nassour, Dindial Ramotar, Sambasivarao Damaraju, John Mackey, Tarek Bismar, Michael Witcher, Moulay A. Alaoui-Jamali

Figure S1. Proteins elution profile of nuclear extract subjected to gel filtration chromatography using FPLC.
Nuclear protein extracts from WT BT-20 cells were loaded on superoseTM 6 FPLC column eluted at a flow rate of 0.25ml/min. All eluents were collected at 0.5 ml/fraction.

Figure S2. pFascin antibody specifically detects the phosphorylated isoform of Fascin.
(**A**) Schematic of CRISPR target site on the *FSCN1* gene. Guide RNA target region is indicated by an arrow. (**B**) Immunoblot verification of BT20 Fascin$^{+/+}$ and Fascin$^{-/-}$ clones (C1 to C6) with antibodies specific for total and phosphorylated Fascin. GAPDH was used as loading control.

Figure S3. Identification of a novel Fascin transcript.
(A) Modified screenshot for the *Fscn1* gene from GTEX portal. The arrow highlights the difference between both transcripts. (B) Protein sequence alignment of full length and truncated Fascin. The box highlights the deleted region.

Figure S4. Ponceau staining of BT-20 nuclear protein extracts.
BT-20 nuclear protein extracts were fractionated using size exclusion chromatography. Samples were then run on an SDS page, transferred on a nitrocellulose membrane followed by Ponceau staining.

Table S1. KEGG pathway classification of the top five pFascin target genes.

Table S2. Distribution of pFascin binding sites

Table S3. Distribution of the clinicopathological data of the breast patients.

Figure S1

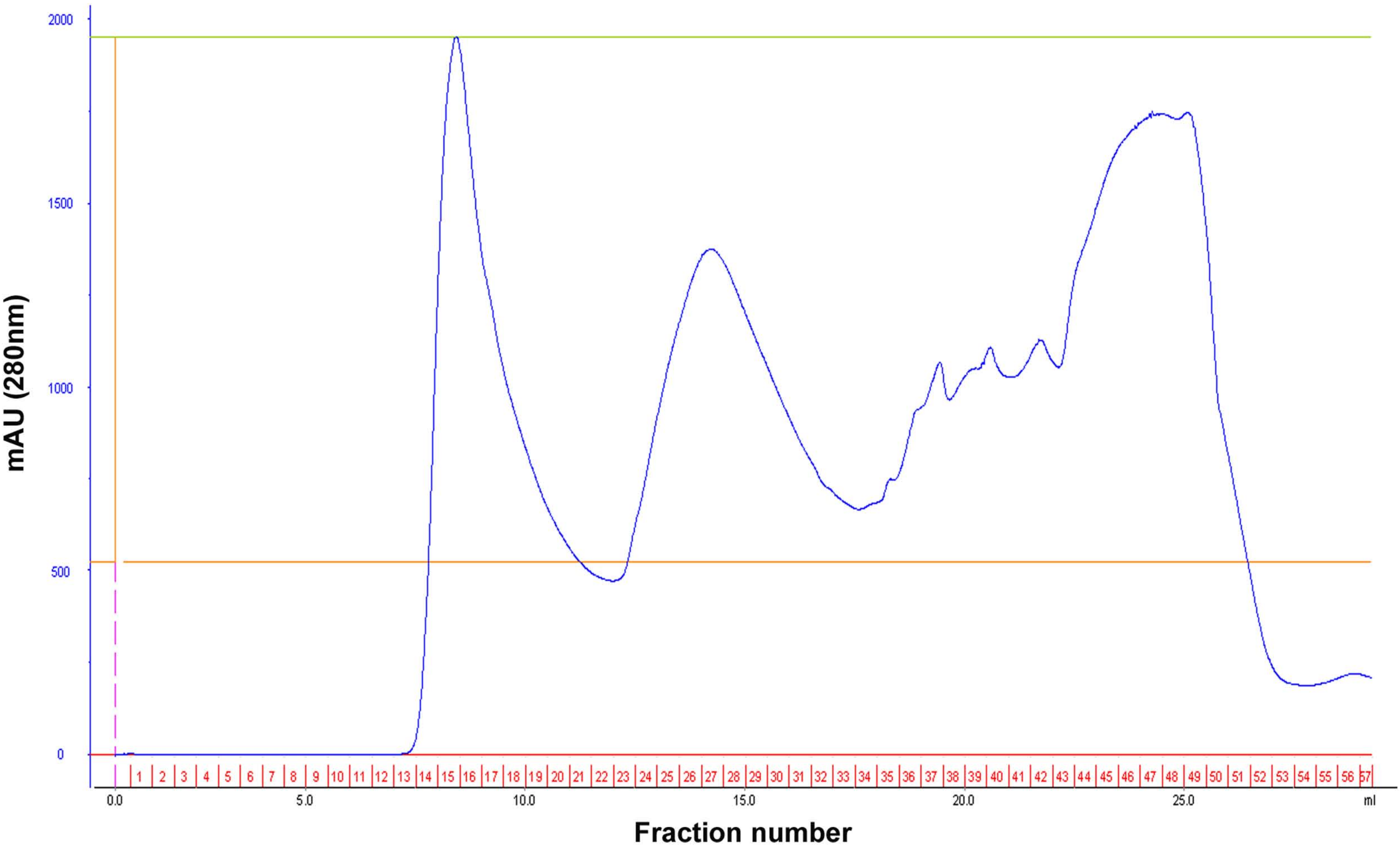

mAU (280nm)
2000
1500
1000
500
0
0.0
5.0
10.0
15.0
20.0
25.0
ml
Fraction number

Figure S2

A

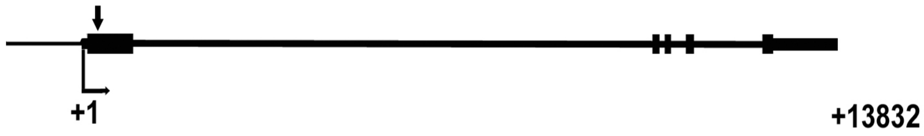
+1
+13832

B

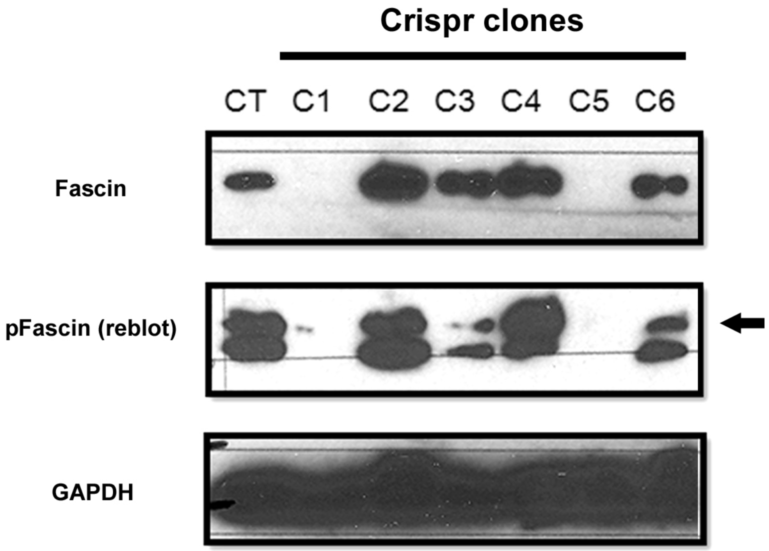
Crispr clones
CT C1 C2 C3 C4 C5 C6
Fascin
pFascin (reblot)
GAPDH

Figure S3

A

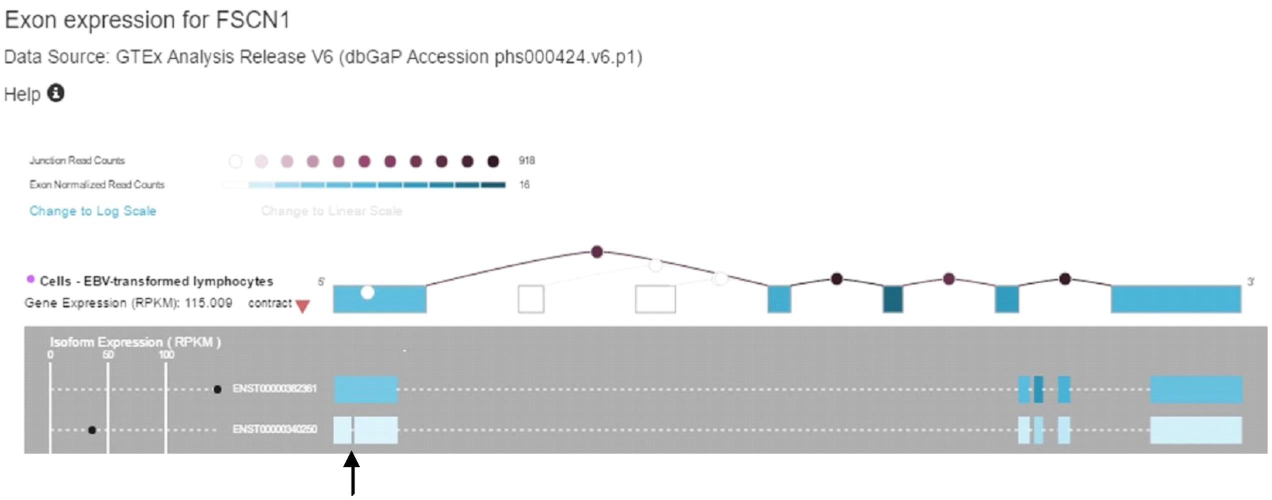

B

```
95.5% identity in 493 residues overlap; Score: 2383.0; Gap frequency: 4.3%

full          1 MTANGTAEAVQIQFGLINCGNKYLTAEAFGFKVNASASSLKKKQIWTLEQPPDEAGSAAV
truncate      1 MTANGTAEAVQIQFGLINCGNKYLTAEAFGFKVNASASSLKK------------------
                ******************************************

full         61 CLRSHLGRYLAADKDGNVTCEREVPGPDCRFLIVAHDDGRWSLQSEAHRRYFGGTEDRLS
truncate     43 ---NHLGRYLAADKDGNVTCEREVPGPDCRFLIVAHDDGRWSLQSEAHRRYFGGTEDRLS
                    ********************************************************

full        121 CFAQTVSPAEKWSVHIAMHPQVNIYSVTRKRYAHLSARPADEIAVDRDVPWGVDSLITLA
truncate    100 CFAQTVSPAEKWSVHIAMHPQVNIYSVTRKRYAHLSARPADEIAVDRDVPWGVDSLITLA
                ************************************************************

full        181 FQDQRYSVQTADHRFLRHDGRLVARPEPATGYTLEFRSGKVAFRDCEGRYLAPSGPSGTL
truncate    160 FQDQRYSVQTADHRFLRHDGRLVARPEPATGYTLEFRSGKVAFRDCEGRYLAPSGPSGTL
                ************************************************************

full        241 KAGKATKVGKDELFALEQSCAQVVLQAANERNVSTRQGMDLSANQDEETDQETFQLEIDR
truncate    220 KAGKATKVGKDELFALEQSCAQVVLQAANERNVSTRQGMDLSANQDEETDQETFQLEIDR
                ************************************************************

full        301 DTKKCAFRTHTGKYWTLTATGGVQSTASSKNASCYFDIEWRDRRITLRASNGKFVTSKKN
truncate    280 DTKKCAFRTHTGKYWTLTATGGVQSTASSKNASCYFDIEWRDRRITLRASNGKFVTSKKN
                ************************************************************

full        361 GQLAASVETAGDSELFLMKLINRPIIVFRGEHGFIGCRKVTGTLDANRSSYDVFQLEFND
truncate    340 GQLAASVETAGDSELFLMKLINRPIIVFRGEHGFIGCRKVTGTLDANRSSYDVFQLEFND
                ************************************************************

full        421 GAYNIKDSTGKYWTVGSDSAVTSSGDTPVDFFFEFCDYNKVAIKVGGRYLKGDHAGVLKA
truncate    400 GAYNIKDSTGKYWTVGSDSAVTSSGDTPVDFFFEFCDYNKVAIKVGGRYLKGDHAGVLKA
                ************************************************************

full        481 SAETVDPASLWEY
truncate    460 SAETVDPASLWEY
                *************
```

Figure S4

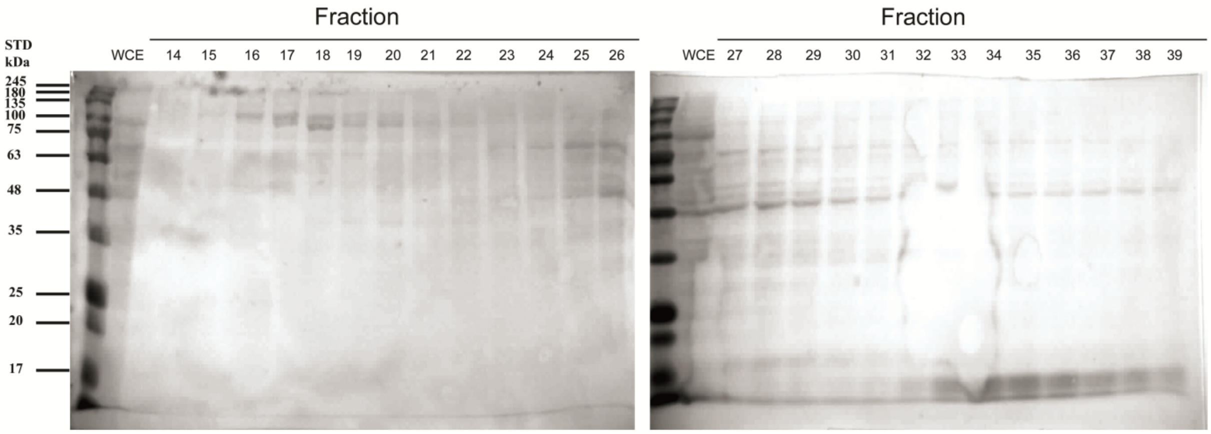
STD
kDa
245
180
135
100
75
63
48
35
25
20
17
Fraction
WCE 14 15 16 17 18 19 20 21 22 23 24 25 26
Fraction
WCE 27 28 29 30 31 32 33 34 35 36 37 38 39

Table S1.
KEGG pathway classification of the top five pFascin target genes

Pathways	Target Genes in Term
Pathways in cancer	209
MAPK signaling pathway	156
Endocytosis	138
Focal adhesion	126

Table S2.
Distribution of pFascin binding sites

Annotation	Peak count	% total
Promoter	3598	15.6680021
Exon	1277	5.5608779
Intron	10732	46.7340185
Intergenic	5287	23.0229925
Other	2079	9.05330082
Total Peaks	22964	100

Table S3. Distribution of the clinicopathological data of the breast patients.

Variable	Category	Paraffin embedded samples n (%)	
		**Benign**	**CA**
Age (years)	Mean	46.6	49.8
Clinical Stage	I	nd	16 (6.8)
	II		191 (81.6)
	III		26 (11.2)
	IV		1 (0.4)
T stage	T1+T2	nd	112 (47.9)
	T3+T4		122 (52.1)
Lymph nodes	pN0	nd	25 (10.8)
	pN+		206 (89.2)
Histological subtype	Ductal	nd	157 (67.1)
	Lobular		13 (5.6)
	Mixed		2 (0.9)
	N/A		59 (25.1)
	Other		2 (0.9)
	Tubular		1 (0.4)
Vascular embolization	No	nd	78 (33.3)
	Yes		156 (66.7)
Dermal lymph node invasion	No	nd	164 (92.7)
	Yes		13 (7.3)
NBR	1	nd	220 (94)
	2		14 (6)

Recurrence	No	nd	164 (70.1)
	Yes		70 (29.9)
Status	Alive	5 (71.4)	189 (80.8)
	Dead	2 (28.6)	45 (19.2)

ND: not determined; Percentages considering cases with complete information
